# Combined one-stage minimally invasive surgery for primary pulmonary carcinoma and mitral regurgitation

**DOI:** 10.1186/s13019-020-1072-y

**Published:** 2020-01-30

**Authors:** Chengfeng Huang, Chao Yang, Jiawen Huang, Qiuying Liao, Xiaoshen Zhang, Shengjie Liao

**Affiliations:** 1 Department of Cardiovascular Surgery, The First Affiliated Hospital , Jinan University, Guangzhou.No.613 Whampoa Avenue, Tianhe District, Guangzhou, China; 2 Department of Cardiovascular Surgery, The First Affiliated Hospital of Guangzhou Medical University, Guangzhou, China; 3 Department of Pharmacy, The First Affiliated Hospital, Jinan University, Guangzhou, China

**Keywords:** Video-assisted thoracic surgery, Minimally invasive surgery, Mitral regurgitation, Adenocarcinoma of lung, Systematic nodal dissection

## Abstract

**Background:**

We report the first successful short-term outcome of one-stage minimally invasive surgery (MIS) mitral valve repair and video-assisted thoracoscopic surgery (VATS) lobectomy.

**Case presentation:**

We report the first successful short-term outcome of combined one-stage video-assisted thoracoscopic surgery lobectomy and minimally invasive surgery in a patient with operable primary right upper lobe adenocarcinoma and mitral regurgitation. Post- operative recovery was uneventful, and follow-up at 6 weeks confirmed an excellent surgical and oncologic outcome.

**Conclusions:**

We think one-stage minimally invasive surgery (MIS) cardiac surgery and video-assisted thoracoscopic surgery (VATS) lobectomy would benefit patients with satisfactory cardiac and pulmonary function.

## Background

Synchronous heart valve disease and primary pulmonary neoplasms are not rare and traditionally surgically approached by staged or simultaneous open strategies that present significant morbidities and surgical risks [1]. We report the first successful short-term outcome of one-stage minimally invasive surgery (MIS) mitral valve repair and video-assisted thoracoscopic surgery (VATS) lobectomy, systematic nodal dissection (SND) in a patient with a synchronously occurring mitral regurgitation and pulmonary adenocarcinoma.

## Case presentation

**Fig. 1:**
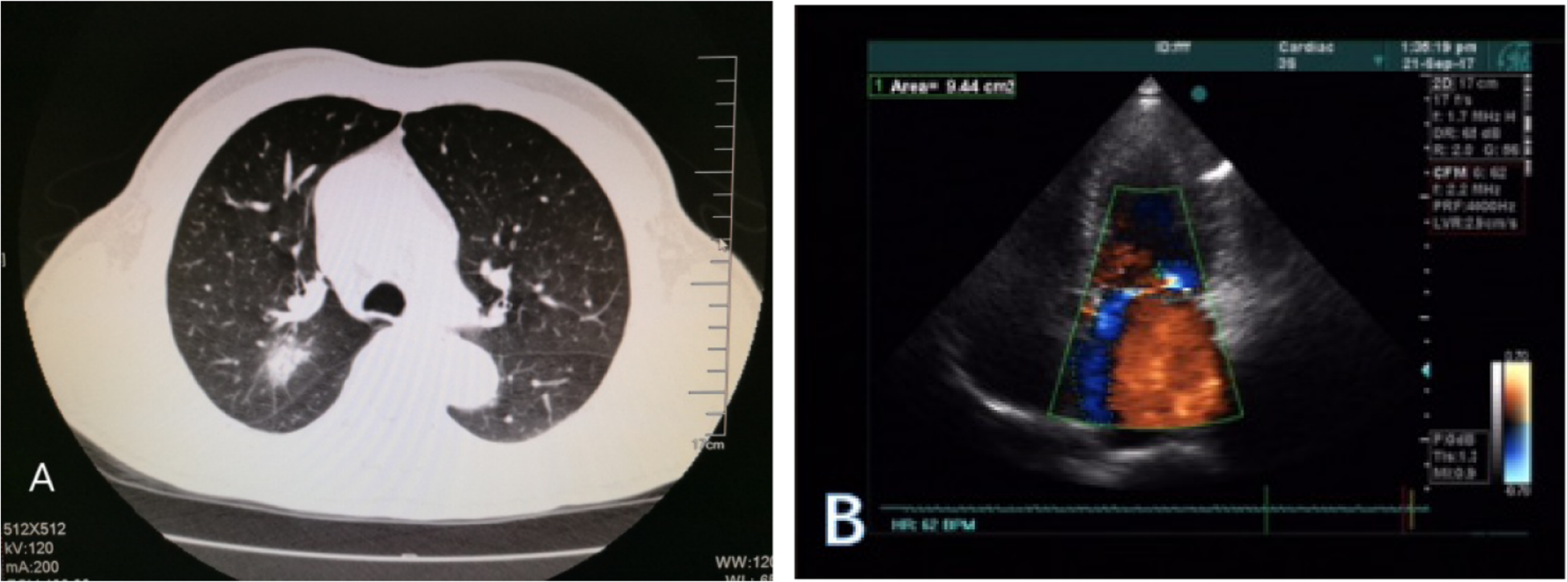
**a** Computed tomography imaging scans identified a right upper lobe apical mass. **b** Transthoracic echocardiographic imaging of Mitral prolapse associated with severe MR

A 67-year-old man identified an enlarging heterogeneous mass in the posterior segment of his right up lobe. The patient had symptoms of shortness of breath after activity and physical examination revealed a grade 3–4 of 6 holosystolic murmur. Echography of the abdomen, Computed tomography (CT) scan of the brain, bone scintigraphy and Positron Emission Tomography (PET) were performed to define the pulmonary malignancy and excluded distal metastases (Fig. 1a), tumor was classified and staged T2N0M0. An electrocardiogram (ECG) revealed examination demonstrated severe prolapse of the posterior mitral leaflet with flail of the middle scallop (P2, P3 segment) (Fig. 1b), the left atrial (LA) and left ventricle (LV) was extensive (LA 46 mm, LV 55 mm), the patient was severe mitral regurgitation with New York Heart Association (NYHA) class II. Lung function tests and coronary angiography were unremarkable. The patient denied family history of related diseases. Patient elected the option of a one-stage ipsilateral VATS and minimally invasive cardiac surgery as definitive diagnostic and therapeutic procedures.

### Surgical technique

**Fig. 2:**
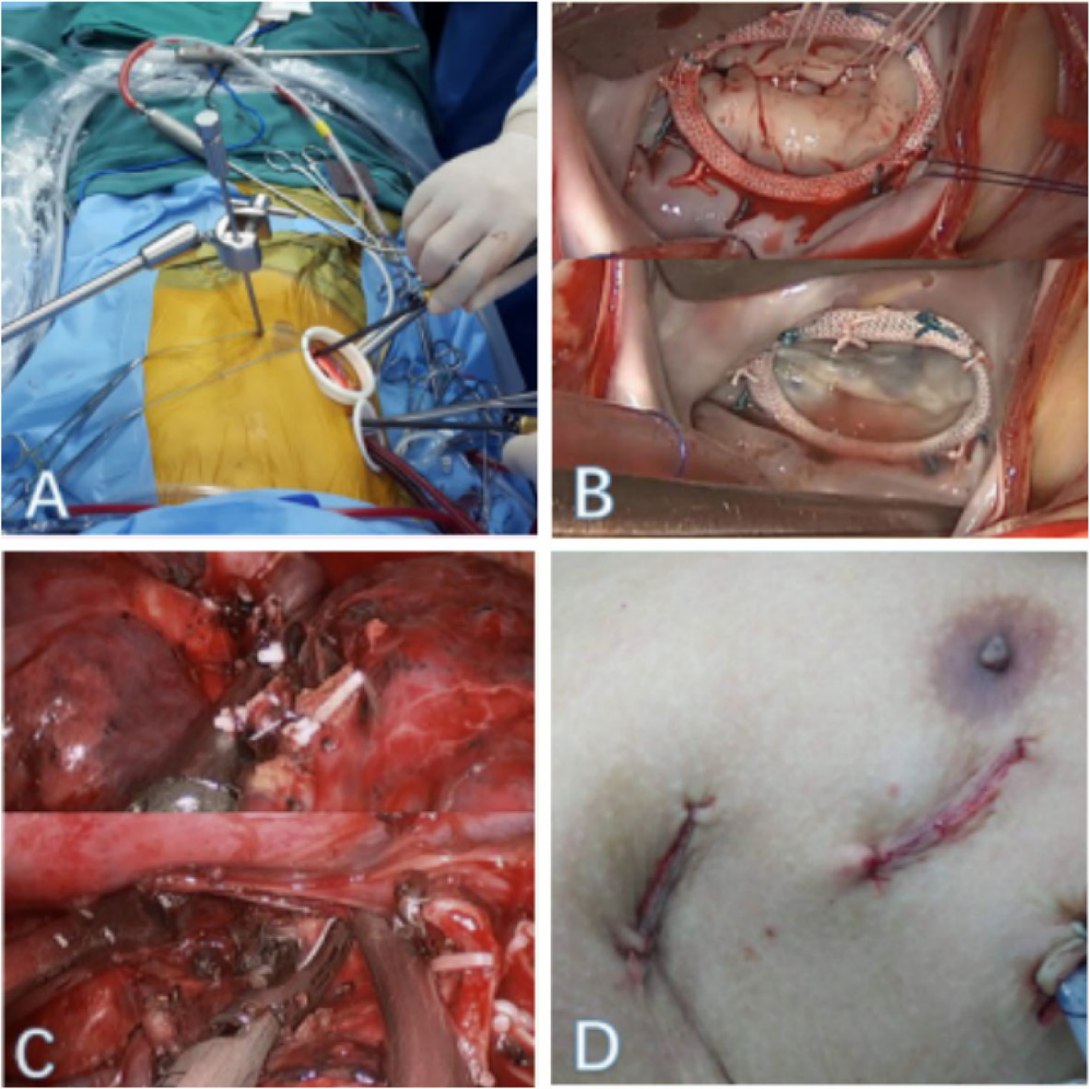
**a** Video-assisted thoracoscopic surgery port incisions (**b**) Minimally invasive MV repair (**c**) Right upper lobectomy and systematic nodal dissection (**d**) Cosmetic result of the incisions

After general anesthesia, double-lumen tube intubation, and insertion of routine monitoring catheters, the patient was positioned supine with the right chest slightly elevated first for MIS of mitral valve (MV) [2]. Cardiopulmonary bypass (CPB) was instituted via femoral arterial and venous cannulation. Anterior axillary ports were inserted in the third, fifth intercostal spaces for assist port and camera port. A right lateral, fourth intercostal space, < 1 cm below and posterior to the nipple, 5 cm mini-thoracotomy was performed (Fig. 2a). An additional venous cannula was inserted in the superior vena cava from the assist port and the aorta was cross clamped with a Chitwood clamp from the same port. Myocardial protection was achieved with mild hypothermia and antegrade delivery of hyperkalemic cold sanguinous cardioplegia (1 L), then the cardioplegia was reinfused every 20 min (500 ml). The left atrium was then opened posterior to the interatrial groove, and a left atrial retractor was used to expose the MV.

Gore-Tex loops were attached to felt pledgets. The correct loop length was estimated by measuring the distance from the papillary muscle to the level of the mitral annulus. The anteroposterior diameter of the anterior MV leaflet and the distance of the commissures for appropriate ring sizing (Edward 28 mm).Valve competency was tested by injecting saline into the left ventricle (Fig. 2b). Heparin reversed with protamine.

The patient was then placed in the left decubitus position. The same intercostal space ports were used for working port. A wedge resection was performed and the frozen biopsy result came back as lung adenocarcinoma. We performed right upper lobectomy [3]. Upper lobe vein was dissected. Stapler Echelon 60 2.5 mm was used for cutting the vein. After the upper lobe bronchus was fully freed by right angle clamp, the bronchus was exposed and identified. The stapler pin was locked into position and the residual lung expansion test was examined, then the stapler was fired and removed. The fissures were completed by means of blunt dissection, cautery and stapler Echelon 60 3.5 mm. The diseased lobe was carefully maneuvered into an endobag. Systematic nodal dissection of stations R2 to R10 was performed by ultrasonic scalpel Harmonicc Ace and lymph node biopsy clamp (Fig. 2c). The inferior lung ligament was freed so that the residual lung could get a well reexpansion.

The patient recovered smoothly after operation, who was free from infusing blood during hospitalization and discharged from hospital on the tenth day after operation. This patient have not complications during follow-up.

## Discussion and conclusions

Although traditionally surgical approached by two-stage pulmonary resection and cardiac surgery was associated with acceptable operative morbidity and mortality [4]. Combine surgery is expected due to advances in surgical and improvements of diagnostic facilities. The one-stage surgery avoids the morbidity, cost, progressive tumor growth, and potential tumor dissemination associated with time delays. Our studies have shown that meticulous hemostasis was carried out and the protamine is fully reversal with heparin before lobectomy, the occurrence of bleeding complications and incomplete lymph node dissection can be reduced. Our successful one- stage VATS oncologic resection and MIS of the MV resulted in a good clinical and cosmetic outcome, rapid patient recovery, and overall patient satisfaction [5] (Fig. 2d).

Even some historical data in the literature suggest that CPB enhance tumor growth and dissemination, because CPB increases the concentations of the free oxygen radicals, which may cause cell damage and inhibit the immune system [6, 7], but cardiovascular disease significantly impacts the morbidity of patients with lung cancer. It is challenging for surgeons to determine the order of the surgery in simultaneous surgery. In this case, the priority of mitral valvuloplasty is to reduce the risk of lung cancer operation.

In our opinion. If the patients with normal respiratory function, it is rational that cardiac operation preceded lobectomy in patients with NYHA II-III, while patients with NYHA I can initially undergo lobectomy [4, 8].

Actually, pulmonary resection through the medium sternotomy is more difficult than the classical thoracotomic approach. But the surgery use a combination of two different approach (median sternotomy and posterolateral thoracotomy), the patient will suffer from more postoperative discomfort. With the development of endoscopic technique using cardiac surgery, MIS cardiac surgery do not increase the risk of surgery. Therefore, one-stage MIS cardiac surgery and VATS lobectomy is benifical for pulmonary resection and not affect the safety of the operation, but also obtain cosmetic result. In addition, avoiding sternum incision can reduce bleeding and free from related complication.

The outcome of this patient who underwent one-stage MIS cardiac surgery and VATS lobectomy was favorable. We believe that some patients can benefit from this combined operation.

## Data Availability

All data generated or analysed during this study are included in the published article.

## Abbreviations

CPB: Cardiopulmonary bypass
CT: Computed tomography
ECG: Electrocardiogram
LA: Left atrial
LV: Left ventricle
MIS: Minimally invasive surgery
MV: Mitral valve
NYHA: New York Heart Association
PET: Positron Emission Tomography
SND: Systematic nodal dissection
TNM: TumorNodeMetastasis
VATS: Video-assisted thoracoscopic surgery
